# NF-κB and its crosstalk with endoplasmic reticulum stress in atherosclerosis

**DOI:** 10.3389/fcvm.2022.988266

**Published:** 2022-09-20

**Authors:** Wenjing Li, Kehan Jin, Jichang Luo, Wenlong Xu, Yujie Wu, Jia Zhou, Yilin Wang, Ran Xu, Liqun Jiao, Tao Wang, Ge Yang

**Affiliations:** ^1^Laboratory of Computational Biology and Machine Intelligence, National Laboratory of Pattern Recognition, Institute of Automation, Chinese Academy of Sciences, Beijing, China; ^2^School of Artificial Intelligence, University of Chinese Academy of Sciences, Beijing, China; ^3^Peking Union Medical College Hospital, Peking Union Medical College, Chinese Academy of Medical Sciences, Beijing, China; ^4^Department of Neurosurgery, Xuanwu Hospital, Capital Medical University, Beijing, China; ^5^China International Neuroscience Institute (China-INI), Beijing, China; ^6^Institute of Cerebrovascular Disease Research and Department of Neurology, Xuanwu Hospital of Capital Medical University, Beijing, China; ^7^Department of Interventional Radiology, Xuanwu Hospital, Capital Medical University, Beijing, China

**Keywords:** NF-κB, endoplasmic reticulum stress, atherosclerosis, unfolded protein response, NLRP3 inflammasome, reactive oxygen species

## Abstract

Atherosclerosis (AS) is a common cardiovascular disease with complex pathogenesis, in which multiple pathways and their interweaving regulatory mechanism remain unclear. The primary transcription factor NF-κB plays a critical role in AS *via* modulating the expression of a series of inflammatory mediators under various stimuli such as cytokines, microbial antigens, and intracellular stresses. Endoplasmic reticulum (ER) stress, caused by the disrupted synthesis and secretion of protein, links inflammation, metabolic signals, and other cellular processes *via* the unfolded protein response (UPR). Both NF-κB and ER stress share the intersection regarding their molecular regulation and function and are regarded as critical individual contributors to AS. In this review, we summarize the multiple interactions between NF-κB and ER stress activation, including the UPR, NLRP3 inflammasome, and reactive oxygen species (ROS) generation, which have been ignored in the pathogenesis of AS. Given the multiple links between NF-κB and ER stress, we speculate that the integrated network contributes to the understanding of molecular mechanisms of AS. This review aims to provide an insight into these interactions and their underlying roles in the progression of AS, highlighting potential pharmacological targets against the atherosclerotic inflammatory process.

## Introduction

The transcription factor NF-κB regulates immunity by controlling the expression of genes associated with inflammation. In mammals, five proteins belonging to the NF-κB family have been identified, NF-κB1 (p50), NF-κB2 (p52), RelA (p65), RelB, and cRel (Table 1). NF-κB exists in the cytoplasm in the form of homodimer (e.g., p50) or heterodimer (e.g., p50/p65) as a family of structurally related proteins (1, 2). It moves into the nucleus to transcribe target genes upon activation. Highly conservative NF-κB plays critical and stable roles in the immune response or embryonic development of many species (3). Recently, some studies have found that the NF-κB signaling pathway is associated with therapy resistance in breast and ovarian cancer (4, 5). On the other hand, accumulating evidence has proved that the NF-κB signaling pathway plays a key role in the development of many inflammatory metabolic diseases such as obesity, insulin resistance, and atherosclerosis (AS) (6).

**TABLE 1:** Components and characteristics of the NF-κB signaling pathway.

Components	Subunits/Precursors	Functions	Structure
NF-κB	NF-κB1 (p50)/p105	Nuclear localization and DNA binding activity; inhibition of transcription	
			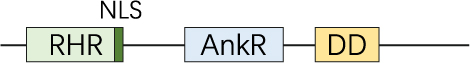
	NF-κB2 (p52)/p100		
			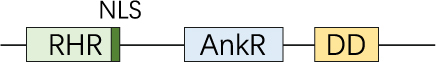
	RelA (p65)	Transcription activity for NF-κB target genes	
			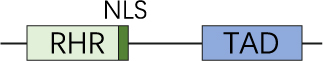
	RelB		
			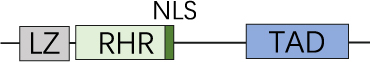
	cRel		
			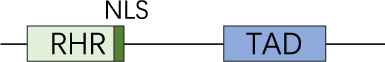
IκB	IκBα	Releasing NF-κB dimer by poly-ubiquitination and degradation	
			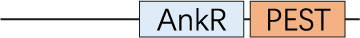
	IκBβ		
			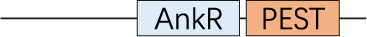
	IκBε		
			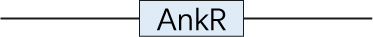
	p100 (IκBδ)	Inhibition of NF-κB by assembling into high-molecular-weight complexes; or being processed into NF-κB subunits	
			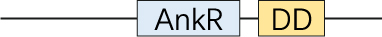
	p105 (IκBγ)		
			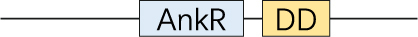
	IκBζ	Modulating NF-κB transcription either positively or negatively	
			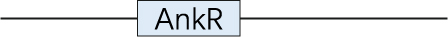
	BCL-3		
			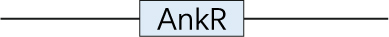
	IκBNS		
			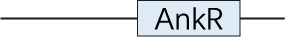
IKK complex	IKKα (IKK1)	Kinase activity	
			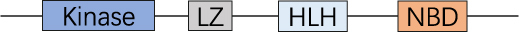
	IKKβ (IKK2)	Kinase activity	
			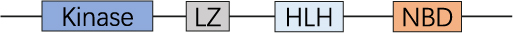
	NEMO (IKKγ)	Regulatory and non-enzymatic	
			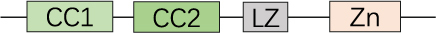

RHR, Rel homology region; NLS, nuclear localization sequence; AnkR, ankyrin repeats; DD, death domain; TAD, transactivation domain; PEST, region rich in proline, glutamate, serine, and threonine; LZ, leucine zipper; Kinase, kinase domain; HLH, helix-loop-helix region; NBD, NEMO-binding domain; CC, coiled-coil domain; Zn, zinc-finger.

The endoplasmic reticulum (ER) is an organelle responsible for protein folding. In the ER, unfolded or misfolded proteins are detected and retained until they are properly folded or degraded. Disturbance in ER protein homeostasis leads to ER stress, activating a specific signaling pathway termed the unfolded protein response (UPR). The UPR is initiated by activation of three ER membrane-bound transducers including inositol requiring enzyme 1 (IRE1), activating transcription factor 6 (ATF6), and protein kinase-RNA like ER kinase (PERK), which alleviates ER stress and helps cells adapt to and survive from ER stress caused by various stimuli (7). However, if the ER stress cannot be resolved, the UPR initiates programmed cell death.

Atherosclerosis is a chronic inflammatory disease contributing to the main pathological basis of ischemic heart disease, myocardial infarct and stroke (8, 9). Increasing evidence has documented that both NF-κB and ER stress closely affect the course of AS, and targeting those pathways may provide new approaches for the treatments against it (10). Herein, some interesting crosstalk in the molecular signaling pathways between NF-κB and ER stress in AS has been reviewed. In this regard, it is reasonable that these links may also be related to AS, which may offer promising opportunities for new strategies against AS.

## Composition and regulation of NF-κB

### The NF-κB signaling

NF-κB activation is initiated from extracellular stimulation signals and is precisely regulated. NF-κB1 (p50) and NF-κB2 (p52) are produced by cleavage of precursors p105 and p100, respectively. In resting cells, NF-κB is kept in the cytosol in its inactive form by binding to IκB (inhibitor of NF-κB) molecule (11). This binding prevents its nuclear localization and transcriptional function by masking the nuclear localization sequence (NLS) at the C-terminus of Rel Homology Region (RHR) (12). RelA (p65), RelB, and cRel contain a transactivation domain (TAD) at the C-terminal end which is responsible for transcribing target genes (Table 1) (13). Thereby, NF-κB dimer consisting of at least one of these three subunits is an active transcription factor, whereas NF-κB containing only p50 and p52 suppresses transcription due to lack of TAD, despite being able to bind to DNA (14).

IκB proteins consist of three groups: the classical IκB proteins, the precursor proteins, and the atypical (nuclear) IκB proteins (14) (Table 1). All of them have an ankyrin repeat sequence (AnkR) for interaction with Rel proteins (2, 15). IκBα, IκBβ, and IκBε belong to the typical group and share the conserved two serine residues at the N-terminal whose phosphorylation regulates the ubiquitination of itself (11). IκBα is associated with dimers of p50-RelA or p50-cRel. It keeps NF-κB in the cytoplasm through an exclusive nuclear export sequence that is exposed when bound to NF-κB. In contrast, NF-κB with IκBβ can locate in the nucleus stably. IκBε and IκBα are found to be the negative feedback regulators of NF-κB back to the cytoplasm (16, 17). NF-κB precursors, p100 (IκBδ) and p105 (IκBγ), also inhibit NF-κB by assembling into high-molecular-weight complexes (18). Phosphorylation of p105 targets it for complete degradation, but it may also promote p105 to be processed into p50 in some cell types (19–21), forming p50-RelA, p50-cRel, or p50 homodimers. Atypical IκB proteins include IκBζ, BCL-3, and IκBNS (Table 1). The most distinct feature of classical IκBs is their extra functions to positively regulate NF-κB (22).

**FIGURE 1:**
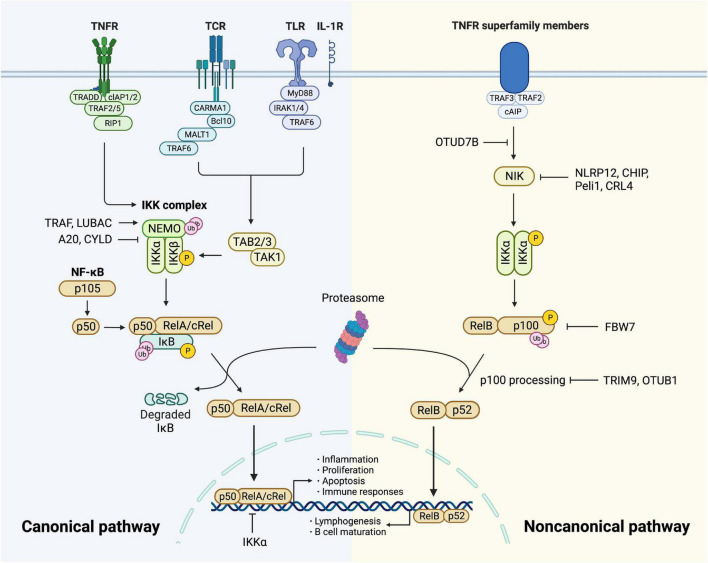
Canonical and non-canonical NF-κB pathway. *The canonical pathway* is induced *via* activation of receptors like TNFR, TCR, TLR, and IL-1R. When TNFR is activated by ligands, it recruits TRADD and drives the assembly of cIAP, TRAF, and RIP1 which is then recruited to NEMO and subsequent formation of IKK complex. TCR recruits CBM complex which is then ubiquitinated by TRAF6, resulting in the activation of TAK1. TLR and IL-1R recruits MyD88 and IRAK1/4, followed by TRAF6 to activate TAK and then IKK complex. TAK1 phosphorylates and activates IKK complex *via* phosphorylation of IKKβ. Then IκB family members phosphorylated by IKK undergo ubiquitin-dependent degradation, resulting in the release of NF-κB dimers. The canonical NF-κB pathway is regulated precisely. IKKα impedes RelA binding to DNA in nucleus. A20 and CYLD destabilize IKK complex *via* their deubiquitination activities. The activity of NF-κB is increased by TRAF- and LUBAC-mediated ubiquitination of NEMO. *The non-canonical NF-κB pathway* is initiated from the stimulation of specific TNFRs, which triggers the recruitment of TRAF3-TRAF2-cIAP and eventually results in stabilization and accumulation of NIK, which is impeded by deubiquitinase OTUD7B. Degradation of NIK is promoted by NLRP12, CHIP, Peli1, and CRL4. NIK phosphorylates and activates IKKα, triggering phosphorylation and ubiquitylation of p100. RelB and p52 generated from p100 constitute NF-κB heterodimer that conducts nuclear translocation and gene transcription. TRIM9 and OTUB1 inhibit p100 processing and FBW7 mediates p100 destruction.

When cells are stimulated by cytokines or pathogen-associated molecular patterns (PAMPs) binding to membrane receptors, signaling cascades initiate and finally converge on the activation of the IκB kinase (IKK) complex (23). The IKK complex consists of three subunits, the catalytic subunits IKKα (IKK1) and IKKβ (IKK2), and the regulatory subunit NF-κB essential modulator (NEMO or IKKγ) (Table 1). IκBs are phosphorylated by the IKK complex, then selectively ubiquitinated by E3 ubiquitin ligase (24), and finally degraded by the proteasome, thus allowing NF-κB translocation to the nucleus. In the nucleus, it is bound to the coactivator molecule to have optimal transcriptional activity (25), leading to gene transcription of growth factors, cytokines, chemokines, adhesion molecules, and other immunoregulatory molecules (Figure 1).

### The activation of NF-κB signaling

Under various stimuli like cytokines, lipopolysaccharide (LPS), UV irradiation, intracellular stresses, and autoantibodies, NF-κB is activated and triggers modification signals. The activation involves two signaling pathways: the canonical and the non-canonical (alternative) pathway (26).

*The canonical pathway* is initiated by tumor necrosis factor receptor (TNFR), T cell receptor (TCR), Toll-like receptor (TLR), and interleukin 1 receptor (IL-1R), leading to rapid but transient NF-κB activation (23, 27). Upon TNF-α binding, TNFR1 drives the assembly of the E3 ubiquitin ligases cellular inhibitor of apoptosis (cIAP) as well as TNFR-associated factor (TRAF) 2 with the protein kinase receptor-interacting protein 1 (RIP1) (28). RIP1 is then ubiquitinated and bound to NEMO (29), forming TGF-β activated kinase 1 (TAK1)-IKK complex. TAK1 phosphorylates and activates IKKβ as well as modification signals. TCR activates NF-κB through the recruitment of CARD11/Bcl10/MALT1 (CBM) complex (30, 31), which is then ubiquitinated by recruiting TRAF6, resulting in the activation of TAK1 as well as IKK (32). TLR and IL-1R initiate signaling through recruiting myeloid differentiation primary response gene 88 (MyD88) directly (33) or indirectly (34) which induces the recruitment of IL-1 receptor-associated kinase (IRAK) 1/4, followed by TRAF6 to activate TAK complex and intracellular signaling cascades (35, 36) (Figure 1). Sequentially, variant modification signals are converged on the activation of TAK1, which activates the IKK complex *via* phosphorylation of IKKβ. IκB family members phosphorylated by IKK undergo ubiquitin-dependent degradation, releasing the canonical NF-κB dimers, predominantly the p50-RelA and p50-cRel (Figure 1). The regulation of the canonical NF-κB pathway occurs at different levels to maintain homeostasis. Firstly, NF-κB transcribes IκBα and IκBε genes to form negative feedback (37). NF-κB activity is also controlled at the transcriptional factor level. For example, IKKα and ubiquitin ligase complex mediate the turnover of RelA (38) and impede its binding to DNA (39). In addition, deubiquitylation of signal molecules upstream of IKK is important in the negative regulation. A20 modifies signaling molecules, especially NEMO to destabilize the IKK complex and down-regulate inflammatory response (40). Tumor suppressor protein cylindromatosis (CYLD) also inhibits the activation of IKK by a similar mechanism (41). IKK inhibitors suppress thrombosis by blocking soluble N-ethylmaleimide-sensitive factor attached protein receptor (SNARE) complex formation and platelet secretion, thus mitigating late-stage plaque development (42). Lastly, canonical NF-κB is positively regulated by ubiquitination of NEMO through TRAF and linear ubiquitin chain assembly complex (LUBAC), which is crucial for IKK activation (43) (Figure 1).

*The non-canonical pathway* is activated slowly and persistently compared to the canonical one. It has a central signaling component, NF-κB-inducing kinase (NIK), equivalent to TAK1 in the canonical pathway. The signaling cascade is based on the stimulation of specific TNFRs by CD40 ligand, B cell-activating factor (BAFF), and lymphotoxin-β (14). The process initiates from TRAF3-TRAF2-cIAP recruitment and ends up with NIK activation (44). NIK phosphorylates and activates IKKα (23, 45, 46), which mediates phosphorylation of p100, triggering its ubiquitylation *via* recruitment of the E3 ubiquitin ligase βTrCP (47–49). The processing of p100 generates p52, resulting in the nuclear translocation of p52-RelB heterodimer. Since the non-canonical activation relies on the generation of p52 from p100, the processing of p100 lies in the key position of regulation. This process is dependent on ubiquitination and phosphorylation, which are regulated by specific ubiquitin E3 ligase and NIK-IKKα axis, respectively. The former includes tripartite motif family 9 (TRIM9) which inhibits NIK-induced and β-TrCP-dependent p100 processing (50). FBW7, also an E3 ligase, exclusively interacts with glycogen synthase kinase 3β (GSK3β) phosphorylated p100 and mediates its destruction (51). OTUB1 is a deubiquitinase that stabilizes p100. As a pivotal node in the non-canonical pathway, NIK has a significant role in NF-κB regulation. Its degradation is promoted by NOD-like receptors family pyrin domain-containing (NLRP) 12 and E3 ligases, CHIP, Peli1, and CRL4 (14). Additionally, OTUD7B, an A20-like protein, deubiquitinates TRAF3 and thus negatively regulates signal-induced non-canonical NF-κB (52) (Figure 1).

Notably, apart from those pathways mentioned above, ER stress has emerged as an important trigger upstream of NF-κB. NF-κB activation mediated by ER stress is dependent on Ca^2+^ efflux and subsequent production of reactive oxygen species (ROS) (13). More mechanisms and interactions will be discussed in detail later in this review.

## The NF-κB and ER stress in atherosclerosis

### Three stages of atherosclerosis progression

Atherosclerosis is a common chronic inflammatory disease characterized by the accumulation of fibrin and lipids in subendothelial space, being a leading cause of cardiovascular diseases, including heart failure, stroke, and claudication (53, 54). AS dominantly occurs in the intima of middle and large-sized arteries, where endothelial cells are exposed to excessive shear stress. Vessel stenosis resulting from atherosclerotic plaque could induce CVD by abolishing blood flow. However, the dominant mechanism linking AS and CVD appears to be the vulnerability of plaque (55). Vulnerable plaque rupture exposes prothrombotic components, triggers the clotting cascade, and leads to atherothrombosis (56). Notably, inflammation is the pivotal cause of plaque progression and vulnerability.

**FIGURE 2:**
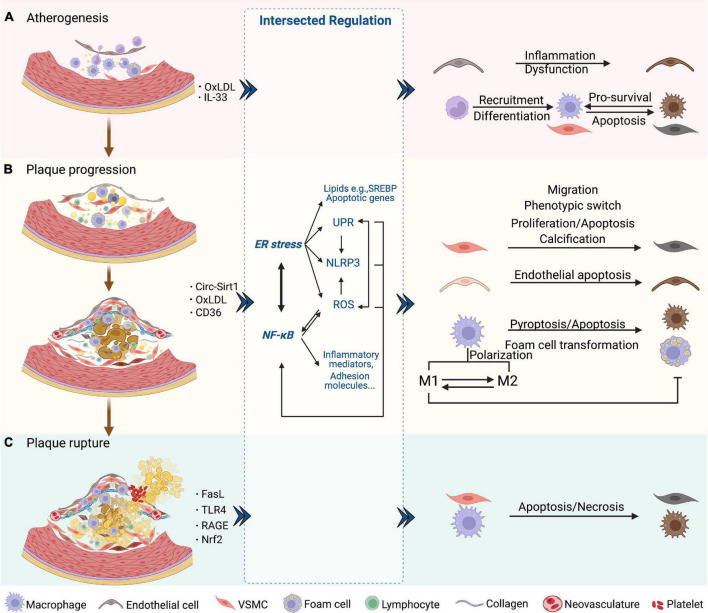
NF-κB and ER stress in three phases of AS. **(A)** Atherogenesis. Endothelial dysfunction as an initial event in atherogenesis is induced by NF-κB and downstream inflammatory mediators. The SREBP pathway is induced by ER stress and aggravates endothelial dysfunction. SREBP- and NF-κB-induced NLRP3 inflammasome contributes to atherogenesis. Chemokines induced by NF-κB attract lymphocytes and trigger endothelial inflammation. NF-κB also promotes the recruitment and differentiation of monocytes by increasing the levels of adhesion molecules and M-CSF of endothelial cells. After differentiated into macrophages, UPR markers are activated, which protects macrophages from ER stress-induced apoptosis. **(B)** Plaque progression. This phase is characterized by foam cell formation, VSMC migration and proliferation, ECM accumulation, and NC formation. ROS/NF-κB regulates the migration and phenotypic switch of VSMCs. Circ-Sirt1 inhibits NF-κB and thus alleviates the progression of AS. Macrophages uptake oxLDL *via* CD36 and this triggers the NF-κB signaling pathways, which promotes the transformation into foam cells. XBP-1 also regulates foam cell formation, endothelial apoptosis and VSMC calcification. Inhibition of ER stress promotes the formation of M1 subtype and subsequent foam cell formation. In macrophages, activated NLRP3 inflammasome causes pyroptosis and apoptosis *via* caspase. **(C)** Plaque rupture. This phase is characterized by less SMCs and collagen, and more lipids and macrophages, which could involve NF-κB-FasL pathway. Macrophages induce plaque rupture by secreting MMPs, which is regulated by TLR4/NF-κB and RAGE/NF-κB signaling. Apoptosis of macrophages and VSMCs is induced by the prolonged ER stress, including PERK and IRE-XBP1. CHOP is also a mediator of apoptosis, vascular remodeling and plaque necrosis, whose expression is promoted by UPR signaling. Nrf2, as a synergistic mediator between NF-κB and ER stress, has an athero-protective role by upregulating some antioxidant enzymes. Additionally, NLRP3 inflammasome-mediated up-regulation of MMPs predisposes plaque to rupture.

Loss of intact endothelial functions occurs at the earliest in atherogenesis, followed by lipid accumulation and fatty streak formation under the endothelial cells. Fatty streak is a reversible lesion that can appear as early as childhood. In this process, multiple molecules mediate leukocyte adhesion, extravasation, migration, chemotaxis, activation, and the formation of foam cells from macrophages by uptake of lipids. Then the nascent plaque generally develops and forms a complex lesion with migration and proliferation of vascular smooth muscle cells (VSMCs), which secrete extracellular matrix such as collagen accumulated in the plaque (57) (Figure 2A). As plaque progresses, a necrotic core containing necrotic material, foam cells, cholesterol crystals, and lipids is formed and developed. Necrotic cores are considered to further promote inflammation, plaque rupture, and thrombosis by storing inflammatory mediators, matrix proteases, and thrombotic molecules (Figure 2B). A fissure of the fibrous cap eliminates the barrier between the tissue factor rich in the lipid core and the coagulation factors in the bloodstream, which triggers a clotting reaction and leads to thrombosis in advanced atherosclerotic lesions (58–61). Finally, the rupture of advanced plaque from the instability of the fibrous cap is primarily determined by the level of interstitial collagen (62). In addition, the disruption of fragile neovasculature in atherosclerotic plaques provides a possibility of sudden plaque progression (63) (Figure 2C).

NF-κB has been regarded as a critical player in atherogenesis over the past decades partly because the genes it transcribed mediate all three phases of AS (64–66). Studies have revealed that IKK/NF-κB signaling promotes atherogenesis and that targeting NF-κB is a treatment strategy against AS and CVDs (67). Nevertheless, sufficient evidence proves that NF-κB activation leads to both protective and destructive outcomes (68). Research suggests that ER stress is associated with various lesions during AS and affects the disease course, which occurs in endothelial cells, VSMCs, and macrophages by integrating protein and lipid metabolism, cell death, and inflammatory responses (69).

Taken together, it is important to figure out how the NF-κB, ER stress-related molecules, and their functional crosstalk intervene in three stages of AS, including atherogenesis (plaque formation), plaque progression, and plaque instability.

### NF-κB and ER stress in early atherosclerotic lesion formation

Endothelial dysfunction, an initial factor in early atherosclerotic lesion formation, is induced by NF-κB and downstream production of inflammatory cytokines, such as IL-6 and TNF-α (70). Regenerated endothelial cells produce a large amount of NO and aggravate inflammatory response, leading to the formation of plaque (71). A recent study found that RIP1 primarily drives inflammatory cells toward activation in early atherosclerotic lesion formation in an NF-κB-dependent manner (10). Moreover, inhibiting cyclooxygenase-2 (COX-2) expression, a downstream gene of NF-κB, dramatically impedes the early evolution of AS (72). CCL20, a chemokine exerting selective attraction to lymphocytes, is upregulated by NF-κB and is strongly associated with vascular endothelial inflammation (73). At this early stage, NF-κB also participates in the production of adhesion molecules in the endothelium, including E-selectin, VCAM-1, and intercellular adhesion molecule-1 (ICAM-1), promoting the recruitment of monocytes (Figure 2A). The effects of NF-κB activation in the early stage of AS are not limited to endothelial cells but also occur in various cell types within the plaque (57). CCL20 is overexpressed in VSMCs of atherosclerotic lesions from coronary artery patients, triggers the inflammatory response, and significantly induces human lymphocyte migration (74). Besides, IL-33 upregulates the macrophage-colony stimulating factor (M-CSF) of endothelial cells through the NF-κB pathway, promoting the differentiation of monocytes (75).

Unfolded protein response activation in endothelial cells can be observed at the very beginning of AS. In athero-susceptible regions, activation of IRE1α and ATF6 is consistent with a high expression of molecular chaperones in ER. Additionally, ATF4 and CCAAT/enhancer-binding protein (CEBP) homologous protein (CHOP) mRNA are highly expressed, along with activated PERK pathway in VSMCs and macrophages at this stage (76) (Figure 2A). UPR activation aims to be a protective response to harmful stress and promotes cell survival in early atherosclerotic lesion formation. For example, UPR is a vital modulator of the sterol regulatory element binding protein (SREBP) pathway to maintain lipid homeostasis and inflammatory response, which are important contributors to atherogenesis (77–79).

### NF-κB and ER stress in plaque progression

NF-κB plays a considerable part in cell survival in addition to well-known pro-inflammatory functions, and the two directions may counteract each other in AS progression (80). Research has suggested that IKKβ deletion increases AS in LDLR deficient mice instead of preventing atherogenesis (68). Given the death of foam cells facilitates the necrotic core due to a defect in clearing accumulated lipids, more attention should be paid to NF-κB’s roles in limiting plaque size other than in pro-inflammation.

NF-κB activation regulates the migration and proliferation of VSMCs, whereas the detailed mechanism is still controversial (81, 82). A study by Mehrhof et al. shows that in a knock-in mouse model expressing the NF-κB super repressor, the proliferation rates of VSMCs did not differ from those in wild-type when stimulated by platelet-derived growth-factor-BB (PDGF-BB) or serum. Further study indicated that VSMC proliferation is regulated by classical mitogenic signaling pathways (MAPK and PI3K pathways) rather than NF-κB (81). These results implicate that NF-κB may essentially play a role in apoptosis and inflammatory responses in VSMCs instead of pro-survival or growth signal in the progression of AS. NF-κB-mediated phenotypic switch of VSMCs involves increased synthesis capacity and decreased contraction capacity, which is closely linked with the accumulation of extracellular matrix and plaque promotion in the progression of AS (83–85). Additionally, blocking ROS/NF-κB/mTOR/P70S6K signaling pathway prevents PDGF-BB-induced VSMC phenotypic switch, multiplication, and migration (83). Circ-Sirt1, as a non-coding RNA (ncRNA) regulator of VSMC phenotype, inhibits NF-κB translocation and binding to target DNA by directly interacting with the p65 subunit in the cytoplasm and facilitating the level of SIRT1 mRNA, respectively, which alleviates neointimal hyperplasia and the progression of AS (85) (Figure 2B). NF-κB activated by autoantibodies is also an important mediator in atherosclerotic lesion growth. 27-kDa heat shock protein (HSP27) in the blood combines with IgG anti-HSP27 auto-antibodies to form an immune complex, which has a role in anti-inflammation and anti-atherosclerosis. HSP27 immune complex activates TLR4/NF-κB signaling and increases the level of anti-inflammatory cytokine IL-10 in macrophages. Moreover, HSP27 immune complex reduces form cell formation by inhibiting oxidized low-density lipoprotein (oxLDL) binding to scavenger receptors (86). In addition, under ER stress, chaperone protein 78 kDa glucose-regulated protein (GRP78) dissociates from ER and moves to the cell surface, resulting in the generation of anti-GRP78 autoantibodies which activate NF-κB and induce the expression of adhesion molecules in human endothelial cells (87).

Generally, macrophages are divided into M1 and M2 subtypes, which have pro-inflammatory and anti-inflammatory effects, respectively. In atherosclerotic plaques, both subtypes are identified and play important roles in plaque progression (Table 2). The disruption of balance is speculated to accelerate foam cell formation and be related to plaque vulnerability (88). M2 subtype is prone to apoptosis as a result of oxLDL toxicity, leading to the accumulation of necrotic material within the plaque (89). NF-κB signaling pathway affects the transition from macrophages to foam cells and its further accumulation in the subendothelial space underlying atherosclerotic disease. In macrophages, oxLDL is taken *via* CD36 and other scavenger receptors and is resistant to the lysosomal enzymes (90). It signals *via* CD36-TLR4-TLR6 and triggers the NF-κB signaling pathway to produce proinflammatory cytokines (91). MiR-216a was found to promote telomerase activation in macrophages *via* the Smad3/NF-κB pathway, contributing to the transition from M2 to M1 (92). Applying fullerene derivatives inhibits the oxLDL-induced differentiation of macrophages into lipid-laden foam cells and plaque progression of apolipoprotein (Apo) E knock-out mice arteries. Mechanically, fullerene derivatives alleviate oxidative stress, inhibit CD36 receptor expression, and reduce TRAF2/NF-κB pathway activation (93).

**TABLE 2:** Differences between M1 and M2 macrophages in atherosclerosis.

	M1	M2
Polarization stimuli	Cholesterol crystals; LPS; Pro-inflammatory cytokines; OxLDLs	TGF-β; IL-10; IL-4; IL-13
Activation pathway	TLR-4 or NF-κB pathway	LXR-α (liver X receptor-α)
Secretion of cytokines	TNF-α; IL-1β; IL-6; IL-12; IL-23	IL-10; TGF-β
Predominant metabolism	Aerobic glycolysis; Fatty acid synthesis; Production of mitochondrial ROS	Oxidative phosphorylation; Fatty acid oxidation (β-oxidation)
Localization	Plaque shoulder and lipid core	Adventitia and areas of neovascularization
Association with plaque stability	Abundant in symptomatic and unstable plaques	Abundant in stable zones of the plaque and asymptomatic lesions
Roles	Occurrence of postapoptotic necrosis after dead cell accumulation; Formation of a necrotic core; Contribution to plaque instability and rupture	Phagocytosis of apoptotic cells and debris; Increase of lipid degradation and prevention of foam cell formation; Resolution of inflammation

Endoplasmic reticulum stress is also a pivotal mechanism regulating plaque progression. Spliced X-box binding protein-1 (XBP-1), a molecule downstream of IRE1 and ATF6, modulates many aspects involved in AS progression, such as macrophage apoptosis, foam cell formation, and IL-8 and TNF-α production. Uncontrolled activation and excessive expression of splicing XBP-1 contribute to endothelial apoptosis and eventually AS evolution, as discovered in the branches and plaques of arteries in ApoE knock-out mice, which may also be related to induction of VSMC calcification (94, 95) (Figure 2B). ER stress is also considered to have an important role in macrophage differentiation. Inhibition of ER stress affects lipid metabolism characterized by an increase in cholesterol efflux, which shifts the M2 subtype to M1 and reduces foam cell formation (96). These studies imply that inhibition of ER stress, which promotes transition toward M1, may decrease foam cell formation, inhibit macrophage apoptosis, and block plaque development.

### NF-κB and ER stress in advanced atherosclerosis

During the last decades, people have been trying to understand the pathophysiology of atherosclerosis, though the precise mechanisms underlying plaque destabilization still remain unclear. In this phase, studies have suggested that macrophages secrete proteases, especially matrix metalloproteinase-9 (MMP-9), to destroy elastin, fibrin, and other matrix proteins that the tension of the fibrous cap comes from, making macrophages an important player in plaque destabilization (97). Several studies support that downregulation of MMP-9 expression in macrophages is mediated by suppressing TLR4/NF-κB signaling, which is associated with attenuation of plaque vulnerability (98, 99). Receptor for advanced glycation end products (RAGE) is a key factor for plaque destabilization in diabetes mellitus, where its downregulation may suppress atherosclerotic plaque development, an effect mediated by NF-κB inhibition (100, 101). Statistical analysis of atherosclerotic lesions from carotid arteries revealed colocalized NF-κB activation and FasL overexpression, and a similar result was also found in peripheral blood mononuclear cells (PBMCs), indicating the NF-κB/FasL pathway may contribute to plaque vulnerability (102) (Figure 2C).

Advanced atheroma provides environmental and molecular bases that trigger ER stress and the UPR. ER-resident molecular chaperone, GRP78/94, and HSP47 are predominantly localized to the VSMC-rich fibrous cap of advanced plaques, suggesting activation of the UPR in VSMCs (103). On the other hand, under prolonged and enhanced ER stress, the activated PERK pathway promotes the level of death effector, and IRE1α/XBP-1 may activate the apoptosis signaling pathway in macrophages and VSMCs at this stage (104, 105). Thin-cap atheroma and ruptured plaques display abundant dead macrophages and VSMCs featuring strongly activated PERK/CHOP which is a mediator of apoptosis on chronic ER stress and a contributor to vascular remodeling and plaque necrosis (106–108) (Figure 2C). The effects of ER stress on the advanced plaque in macrophages are further demonstrated in AS-prone mice lacking CHOP, which shows blockage of macrophage apoptosis and inhibition of necrotic core formation (107, 109, 110).

## The molecular interrelated roles of ER stress and NF-κB in atherosclerosis

Various pathological factors which activate NF-κB, such as ROS, lipids, TLR ligands, and some cytokines (e.g., TNF-α and IL-1), disrupt ER homeostasis and activate the UPR, leading to the situation called ER stress (111). Of note, this relationship is not likely one-sided. There are several potential avenues through which ER function also affects inflammatory signaling. And their interplay constitutes the pathological basis of many inflammatory and metabolic diseases, including AS (112–114). The ER stress is initiated with the dissociation of chaperone proteins such as GRP78/Bip and GRP94 with the ER stress sensor proteins (IRE1α, PERK, and ATF6), which leads to UPR activation. Chaperones also directly participate in subsequential UPR and NF-κB signaling. ATF6 and IRE1α pathways promote the transcription of the ER chaperones, which is necessary for the alleviation of the misfolded proteins to restore homeostasis (115). GRP78 is a member of the chaperone HSP70 family which is closely relevant to the endothelial dysfunction in the development of AS, with a fundamental role in protecting protein stabilization and also in anti-inflammation (116). Note that HSP70s suppress the expression of inflammatory cytokines *via* inhibiting the NF-κB. HSP70s stabilize the IκB complex through its binding and block IKK kinase activity and further NF-κB mediated transcription (117, 118).

**FIGURE 3:**
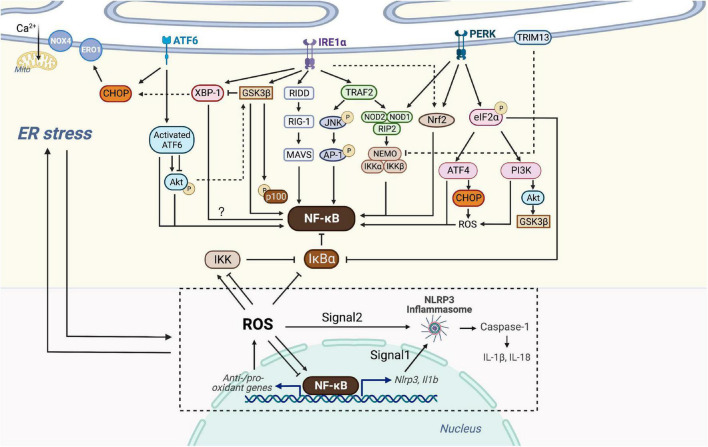
Crosstalk of NF-κB and ER stress. Three branches of UPR (IRE1α, PERK, and ATF6) of ER stress are able to intersect with NF-κB. Activated IRE1α recruits TRAF2, which activates JNK and then AP-1 or associates with IKK probably *via* NOD1/2 and RIP2. IRE1α is also linked with the RIDD/RIG-I/MAVS pathway and GSK3β to activate NF-κB. IRE1α oligomerization increases XBP-1 expression which might be associated with decreased NF-κB expression, but GSK3β activation inhibits IRE1α-dependent XBP-1 splicing. PERK branch can induce NF-κB activation essentially by translation attenuation of the free IκBα mediated by phosphorylated eIF2α. Additionally, PERK-eIF2α could also contributes to inflammation *via* ATF4 or PI3K-Akt pathway. Both of NOD1 and Nrf2 could be activated by PERK and IRE1, but Nrf2 has both positive and negative effects on NF-κB, dependent on cellular circumstances. Through the ATF6 branch transient phosphorylation of Akt activates NF-κB, whereas ATF6 activation could inhibit Akt-GSK3β and enhance NF-κB signaling. Additionally, ER E3 ubiquitin ligase, TRIM13 ubiquitylates NEMO and prevents nuclear translocation of NF-κB. CHOP could be activated by all three branches of UPR, causing ROS-mediated ER stress and NF-κB inhibition or activation. ER stress-induced NLRP3 inflammasome is dependent on NF-κB and UPR activation. Signal 1 of NLRP3 inflammasome activation is transcriptional upregulation of NLRP3 along with pro-IL-1β provided by NF-κB. Signal 2 is a posttranscriptional modification which can be provided by ROS. NF-κB controls the levels of ROS by regulating anti-oxidant and pro-oxidant genes, and ROS in turn inhibits or enhances the DNA binding activity of NF-κB itself, depending on modifications of NF-κB. ROS also regulates the IKK complex and phosphorylates IκBα. ROS produced by Nox4 transduces ER stress signals to the UPR to maintain homeostasis, whereas ROS produced by ERO1 or mitochondrial damage leads to cell death. ROS, NF-κB, NLRP3 inflammasome and the production of IL-1β and IL-18, in turn, trigger chronic ER stress.

Three branches of UPR (IRE1α, PERK, and ATF6) of ER stress have been reported to have crosstalk with many inflammation-related signaling, including the NF-κB pathway. For example, activated IRE1α and recruited TRAF2 activate JNK, inducing the production of IL-6 and TNF-α by phosphorylation of AP-1 and consequent NF-κB activation. ER stress induces TRAIL receptor activation which leads to apoptosis through the FADD/caspase-8 pathway, or alternative production of inflammatory cytokines through NF-κB activation (119–121). However, ER stress can also lead to inhibition of inflammation. The ER E3 ubiquitin ligase TRIM13 ubiquitylates the IKK regulatory subunit NEMO, blocking the degradation of IκBα, which consequently inhibits NF-κB translocation into the nucleus (122). Hence, it makes sense to unravel the exact molecular mechanisms of ER-stress-induced inflammation. Here we focus on how ER stress intersects with NF-κB through various inflammatory signaling pathways to form this integrated network (Figure 3).

### Crosstalk through IRE1α

Several signal cascades have been discovered in the NF-κB activation *via* IRE1α kinase activity. Activated IRE1α kinase recruits TRAF2, which associates with IKK and degrades IκBα to activate NF-κB (123). It is confirmed in endothelial cells that LPS induces ER stress and overproduction of IL-6 and MCP-1 through IRE1α/NF-κB pathway, resulting in endothelial dysfunction (124). Moreover, Keestra et al. found that *Brucella abortus* infection triggered ER stress and induced inflammation and IL-6 production in a TRAF2, nucleotide-binding oligomerization domain-containing protein (NOD) 1/2, and RIP2-dependent manner, providing a novel connection between ER stress and NF-κB activation (125). IRE1α is also linked with the RIDD/RIG-I pathway upon encountering viral RNAs, which induces an inflammatory response through MAVS and downstream NF-κB (126). In addition, IRE1α oligomerization generates spliced XBP-1 mRNAs that are translated into potent transcription factors (127). Increased XBP-1 expression contributes to the secretion of myeloperoxidases, TNF-α, IL-6, and IL-1β, and is negatively correlated with NF-κB expression in the colon (128). Also, ER stress-induced IRE1α activation mediates GSK3β activation and subsequent IL-1β gene expression (129). XBP1s K60/77R mutation, preventing the ubiquitination and proteasome-degradation of XBP1s, mimics the constitutive activation of IRE1α elevated, and results in the elevated GSK3β phosphorylation (130). *In vivo* and *in vitro* studies have confirmed that GSK-3β activation is involved in NF-κB activation, suggesting crosstalk between ER stress and NF-κB through IRE1α/GSK3β pathway (131, 132).

Interestingly, GSK3β activation inhibits IRE1α-dependent XBP-1 splicing, and they differentially regulate proinflammatory cytokine gene expression, indicating complex signaling crosstalk in inflammatory pathways (Figure 3).

### Crosstalk through PERK

Protein kinase-RNA like ER kinase branch can induce NF-κB activation essentially by translation attenuation, including the free IκBα, mediated by phosphorylated eIF2α. Zhang et al. observed that anti-dsDNA antibodies activate NF-κB and upregulate various inflammatory cytokines through PERK-eIF2α-ATF4 (133). Besides, a recent study has shown that thapsigargin-induced PERK activation along with the inositol triphosphate receptor (IP3R)-mediated calcium flux makes cells more responsive to *Salmonella typhimurium* through the NOD1-stimulated NF-κB activation and subsequent inflammatory response (134). Nuclear erythroid-related factor 2 (Nrf2), a transcription factor mainly activated by PERK and IRE1, also plays a pivotal role in the crosstalk between UPR and NF-κB. Studies on the linkage between Nrf2 and autophagy have shown that Nrf2 activates IKK and subsequent NF-κB by enhancing the expression of p62, which explains NF-κB-dependent autophagy activation (135–137). Complex interrelation indicates that Nrf2 influences NF-κB both positively and negatively due to various circumstances. For instance, studies on Nrf2 knock-out mouse embryo fibroblasts have shown increased activity of IKKβ and degradation of IκBα (138). Moreover, the increase of Nrf2 activity in patients with lupus nephritis prevents p65 activation by accumulating glutathione. Increased heme oxygenase-1 (HO-1), a product of the Nrf2 target gene, inhibits adhesion molecules such as E-selectin and vascular cell adhesion molecule-1 (VCAM-1) expressed in endothelial cells *via* NF-κB downregulation (139). Additional experiments have implicated the PERK-eIF2α signaling as a contributor to inflammation *via* the JNK and PI3K-Akt pathway, but the detailed interaction with NF-κB has not been well defined (140) (Figure 3).

Since Nrf2 serves as a platform of interrelation between NF-κB and ER stress (Figure 3), special attention has been paid to this transcription factor to better define its possible contribution to oxidative stress of the vulnerable plaque (141) (Figure 2C). The expansion of the necrotic core and the disruption of the plaque are largely determined by the accelerated number of apoptotic cells and phagocytic clearance defect. Nrf2 not only upregulates the expression of different antioxidant enzymes but also regulates mitochondrial ROS production through NADPH oxidase (Nox) activity. Though most studies have demonstrated the protective roles of Nrf2 against AS, several studies have revealed that it might play antagonistic roles, both preventing and enhancing AS. Studies found that laminar blood flow stimulates the anti-atherogenic activation of Nrf2, whereas oscillatory blood flow promotes the opposite effect (142). Nrf2 in bone marrow-derived cells promotes plaque progression in ApoE knock-out mice (143), while early AS is aggravated in LDLR knock-out mice with Nrf2-deficient macrophages (144). The positive atherogenic role of Nrf2 appears to be implemented by IL-1 release and by promoting foam cell formation through the expression of the CD36 scavenger receptor (145, 146).

### Crosstalk through ATF6

As one of the UPR branches, ATF6 also plays a nonnegligible role during ER stress and in its crosstalk with NF-κB. However, Yamazaki et al. have confirmed that subtilase cytotoxin-triggered rapid cleavage of molecular chaperone GRP78/BiP (78-kD glucose-regulated protein/immunoglobulin heavy chain binding protein in pre-B cells) leads to Akt phosphorylation mediated by ATF6, contributing to downstream NF-κB activation (147). Recently, another study reported that the decrease of ATF6 expression induced by miR-149 might attenuate inflammation and apoptosis through NF-κB and Akt signaling cascades (148). In addition, *in vitro* study showed that ATF6 activation induced by chemical agents inhibits Akt/GSK3β and increases NF-κB activity, thus improving the pro-inflammatory effect of TLR4 in ER-stressed macrophages (149). Despite representing unique signaling cascades, ample evidence has indicated that the UPR and NF-κB may converge on nuclear transcription factors, such as ATF3/4/6α, CHOP, and XBP-1 (150) (Figure 3). Taken together, the UPR has crosstalk with NF-κB at various levels, which offers perspectives on the adjustment of cellular stress responses and therapeutic application in the future.

### Crosstalk through NLRP3

The NLRP3 inflammasome is a multi-protein complex that recognizes PAMPs or damage-related molecular patterns (DAMPs) and activates the protease caspase-1, leading to pyroptosis and the formation of mature IL-1β and IL-18 to mediate the inflammatory response (151). NLRP3 inflammasome connects lipid metabolism and inflammation because it is activated by crystalline cholesterol and oxLDL in plaques of AS, making it a possible player in the development of AS. In general, transcription and modification signals of the NLRP3 are necessary for its function. The former is provided by the binding of LPS to TLR4, resulting in NF-κB activation and consequent transcription of NLRP3 and IL-1β precursor (152). The modification signals occur after transcription, one of which is BRCA1/BRCA2-containing complex subunit 3 (BRCC3)-mediated deubiquitination (153–155). Though the exact process remains unanswered, it is considered that the activation of the NLRP3 inflammasome is possibly associated with factors such as K^+^ outflow, ROS, Ca^2+^ flux, and lysosomal rupture, all of which can provide modification signal (156). Notably, these mechanisms contribute to signal one by activating NF-κB through ROS production. Hu et al. demonstrated that in LPS-induced endometritis in mice, NLRP3 inflammasome is activated *via* ER stress-associated pathway, along with increased NF-κB and ROS (157). In LPS-induced liver injury, NF-κB and the NLRP3 inflammasome activation along with cytokine production such as TNF-α, IL-1β, and IL-18, in turn, contribute to chronic ER stress to form negative feedback (158). A recent study has observed that the ER stress-induced NLRP3 inflammasome is dependent on NF-κB activation and pro-inflammatory cytokine secretion, which is linked to the pathogenesis of atrial fibrillation and can be potentially targeted in cardiac tissue (159). Nevertheless, evidence has revealed that UPR is not indispensable for inflammasome activation (160). Since UPR is involved in NF-κB activation and ROS production, which are related to the activation of the NLRP3 inflammasome, these controversial results call for further insight into UPR pathways as inflammasome mediators (Figure 3).

Atherosclerosis has been considered an inflammatory and lipid metabolic condition, and since the NLRP3 inflammasome is activated by lipids such as crystalline cholesterol and oxLDL, it presumably combines different pathological bases of AS. The NLRP3 inflammasome and subsequent caspase-1 activation cause pyroptosis in macrophages after uptake of oxLDL and might contribute to the progression of atheroma (161, 162). On the other hand, the NLRP3 inflammasome induces macrophage apoptosis *via* caspase-8 activation (163), though to what extent this pro-apoptotic function protects against AS development is still unanswered (Figure 2B). IL-1β and IL-18 produced by the NLRP3 inflammasome increase the expression of many endothelial molecules such as MCP-1, VCAM-1, and IL-8, involving inflammatory cell adhesion, chemotaxis, recruitment, and activation (164). Moreover, the NLRP3 inflammasome promotes plaque instability and subsequent thrombogenesis (165). Blocking NLRP3 signaling reduces the production of pro-inflammatory cytokines in ApoE knock-out mice and contributes to plaque stabilization by reducing macrophages and lipids as well as increasing SMCs and collagen (166).

Although numerous studies have reported the impact of NLRP3 inflammasome on the progression of AS, evidence has suggested it is not as important as we have thought. *In vivo* NLRP3 inflammasome is not critically implicated in AS progression, infiltration by macrophages, and stability of plaques (167). Research also supported that NLRP1 is more likely to be a critical factor for the initiation of endothelium inflammation (168). In addition, JNK1 and apoptosis signal-regulating kinase 1 (ASK1) contribute to inflammasome activation and caspase-8-mediated macrophage apoptosis, though whether this JNK1/ASK1/caspase-8-dependent apoptosis is directly mediated by NLRP3 inflammasome is uncertain (155). The identified pro-apoptotic activity of NLRP3 inflammasome might produce an anti-atherogenic effect, which could partly explain its controversial functions in AS.

### Crosstalk through reactive oxygen species

The relationship between NF-κB and ROS is not one-sided. ROS is a key route linking the two events. Firstly, ROS activates or inactivates the IKK complex in different cell types (169). Often ROS alternatively phosphorylate IκBα, which may result in the release and activation of NF-κB (169, 170). Also, ROS may inhibit or enhance the DNA binding affinity of NF-κB itself, depending on different forms of modification in NF-κB heterodimers (171, 172). Another manner in which ROS interacts with NF-κB is the crosstalk between JNK and NF-κB, preventing persistent JNK activation and promoting cell survival (173).

As to the interactions between ER stress and ROS, it is proved that ROS plays both positive and negative roles during ER stress and in determining cell fate (174). Upon being produced by Nox4, an ER-resident oxygen-sensing enzyme, ROS acts as a signaling intermediate to transduce ER stress-related signals to the UPR, resulting in the correction of the unsteady state. However, ROS as a pro-inflammatory stimulus can further exacerbate inflammation after the UPR activation (111). On the other hand, if ER stress persists, delayed expression of the transcription factor CHOP leads to induction of ER oxidase 1 (ERO1) to produce ROS. Meanwhile, mitochondria exaggerate ROS production stimulated by the Ca^2+^ released from ER. Both contribute to a secondary increase in ROS, generally leading to cell death. Therefore, ROS lies both upstream and downstream of the UPR, making the network composed of ER stress, ROS, and NF-κB more complex than we have imagined.

Substantial evidence indicates that ROS is a central factor through which ER stress functions cooperatively with NF-κB in inflammation and other cellular processes. Li et al. observed that recombinant *Treponema pallidum* protein regulates the ROS/NF-κB pathway through ER stress. PERK induces the activation of the NF-κB and JNK pathways, leading to the production of IL-1β, IL-6, and IL-8 by macrophages (175). In another study, NF-κB signaling is activated by phosphoinositol 3-kinase δ (PI3Kδ) through ER-associated ROS and RIDD-RIG-I activation, which may induce severe airway inflammation and hyperresponsiveness (176). In human lung cancer cells, it is observed that a CHOP activator induces necrotic cell death *via* ROS-mediated ER stress induction and unusual NF-κB inhibition (177) (Figure 3).

The contribution of ROS to AS has been well investigated. ROS causes endothelial dysfunction (178), atherogenesis (179), and LDL oxidation (180). OxLDL has pro-inflammatory effects and participates in the phenotype switching and apoptosis of macrophages and VSMC in the AS progression (181, 182). ROS is positively related to atherosclerotic risk factors, such as diabetes and hypertension, etc. *In vivo* studies of the animal model have also shown that anti-oxidant treatments delay or prevent AS (183), suggesting the aggravating role of ROS in AS. A recent study has demonstrated that nicotine-induced autophagy and subsequent phenotypic transition of VSMCs accelerate AS, which is partly mediated by the nAChRs/ROS/NF-κB signaling pathway (184). In addition, in cultured VSMCs, chicoric acid impeded PDGF-BB-induced VSMC phenotypic alteration, proliferation, and migration mechanistically by blocking ROS/NF-κB/mTOR/P70S6K pathway (83) (Figure 2B). However, the diverse effects of ROS have been reported in AS. Nox4 is a major ROS-producing NADPH oxidase and is widely expressed in VSMCs. Its endothelial-specific overexpression increases ROS level, promotes aging, and makes cells susceptible to apoptosis, resulting in aggravated AS lesions in animals (185–187). Of note, it is also found in several mice models that Nox4 knock-out promotes initial plaque formation (188). Unlike Nox4, another NADPH oxidase Nox2 overexpressing leads to atherogenic rather than protective consequences (189), highlighting the controversial roles of Nox-dependent ROS in AS. The crosstalk between ER stress and ROS may be pivotal to understanding the controversial effect of ROS. Nox4 but not Nox2 selectively phosphorylates eIF2α, the downstream PERK arm of UPR, thus providing a direct route for integrating ROS and ER stress. In addition, Nox4 is central to a signaling feedback loop of Rho/Ras GTPase and ER stress. RhoA activation occurs on ER surface in response to UPR and further promotes Nox4-dependent ROS production (190). Nox4-generated oxygen inactivates ER calcium transporter SERCA (Sarcoplasmic Reticulum Ca^2+^ ATPases) and causes calcium-calmodulin-dependent activation of RasGRF1/2, which further mediates the UPR activation (191). Thus, ROS is more than a marker of oxidative stress, but plays two opposite roles in ER stress (restoration of homeostasis or apoptosis) and involves inflammation and cell growth. These data emphasize the controversial effects of ROS and careful considerations in Nox inhibitor development aiming to reduce ROS levels. It is challenging for Nox4 inhibitor development to retain the ER stress inhibition activity and the athero-protective function of Nox4. Given the diverse signaling roles served by Nox4, more specific Nox inhibitors targeting Nox1 and Nox2 while excluding Nox4 could be an optimal treatment strategy (174).

## Pharmacological targeting of NF-κB and ER stress in atherosclerosis

Innovation of prevention and treatment strategies against AS is still a pressing mission given being the leading cause of mortality and morbidity in developed and developing countries. Despite various interactions between ER stress and NF-κB, whether and to what extent these mediator molecules play a role in AS remains unanswered. Conceptually, several existing pharmacological targeting on UPR, ROS, NLRP3 inflammasome or other crossroads between ER stress and NF-κB could potentially influence both of them and impede the progression of AS. Herein, we focus on NF-κB inhibitors, UPR inhibitors, ROS-interfering molecules, natural compounds, and some ncRNAs with anti-atherogenic protective effects, targeting ER stress and/or NF-κB, which are attractive potential therapeutic strategies for AS (Table 3).

**TABLE 3:** NF-κB and/or ER stress modulators in experimental atherosclerosis and associated disease models.

Category	Modulator	Disease	Model	Pharmacological effect	References
NF-κB inhibitors	BAY 11-7082	Cancer; inflammatory diseases; neurological diseases	LPS-stimulated RAW264.7 macrophages	Inhibition on the translocation of p65, AP-1, IRF3, and STAT-1; inhibition of the phosphorylation of ERK, p38, and JAK-2	(192)
			Imiquimod cream-induced rat model of psoriasis-like dermatitis	Reduction of pNF-κB, NLRP3, TNF-α, IL-6, IL-1β, IL-23, and phosphorylated STAT3	(193)
			*In vitro* and *in vivo* xenograft model of oral cancer	Reduction of OSCC cell viability and of NLRP3 inflammasome, caspase-1, IL-1β, and IL-18 expression; increase of Bax, Bad, and p53 expression; reduction of Bcl-2 expression	(194)
	Pyrrolidine dithiocarbamate (PDTC)	Inflammatory disease especially AS	Rat aortic SMCs	Activation of p38 MAPK and JNK; VSMC growth inhibition	(195)
			ApoE knock-out mice	Blockade of NF-κB; down-regulation of IL-18, IL-18Rα, CD36, and MMP-9; promotion of plaque instability	(196)
	IMD-0354	Cancer; inflammatory diseases; cardiovascular diseases	Organ culture of rat mesenteric arteries with removed endothelium	Inhibition on the up-regulated ET (B2) receptor expression and NF-κB activation	(197)
			Melanoma A375 cells and skin epidermoid carcinoma A431 cells	Inhibition of glutamine uptake; attenuation of mTOR signaling; modulator of cell cycle, DNA damage response and UPR/ATF4/CHOP	(198)
UPR inhibitors	Sirtuin 1 (SIRT1)	Cardiovascular diseases	Cardiomyocytes and adult-inducible Sirtuin 1 knock-out mice	Protection against ER stress-induced apoptosis; NAD^+^-dependent deacetylase, alleviating activation of the PERK/eIF2α branch of the UPR	(199)
	Irisin	Metabolic disorders and AS	OxLDL-induced RAW264.7 macrophages	Alleviation of the apoptosis by inhibiting the PERK/eIF2α/CHOP and ATF6/CHOP ER stress signaling pathways	(200)
	STF-083010 and 4μ8C	Metabolic disorders; AS; cancer	Tunicamycin-treated or high-fat diet fed BI-1 knock-out mice	Reduction of atherosclerotic plaque size; inhibition of IRE1α RNase activity, lipid-induced mtROS production, NLRP3 inflammasome activation, and consequent secretion of IL-1 and IL-18	(205)
ROS-interfering molecules	(E/Z)-BCI hydrochloride	Cancer; inflammatory diseases	LPS-activated macrophages	Inhibition on LPS-triggered inflammatory cytokine production; affecting macrophage polarization to an M1 phenotype; decrease of ROS production; inhibition on phosphorylation and nuclear expression of p65; elevation of Nrf2 levels	(206)
	Dihydrolipoic Acid	Inflammatory and neurological diseases	LPS-induced sickness behavior rat model	Increase of the expression of ERK, Nrf2, and HO-1; decrease of the ROS generation levels and the expression of NLRP3, caspase-1, and IL-1β	(207)
	LGH00168	Cancer	A549 human NSCLC xenograft mice	CHOP activator; induction of necroptosis *via* ROS-mediated ER stress and NF-κB inhibition	(177)
Natural compounds	Baicalin	Cardiovascular diseases; cancer	Neonatal rat cardiomyocytes	Protection from ER stress-induced apoptosis; targeting the CHOP/eNOS/NO pathway	(210)
	Quercetin	Cancer	Glucosamine- induced RAW264.7 macrophages	Prevention of apoptosis and lipid accumulation by inhibiting ER stress; decrease of CHOP and GRP78 expression; increase of ATF6 expression, activated JNK and caspase-12	(211)
	Resveratrol	Cancer; cardiovascular diseases; infection	Isoproterenol-induced rat cardiomyocytes	Inhibition of cardiomyocyte hypertrophy and apoptosis by suppressing ER stress; decrease of GRP78, GRP94, and CHOP expression; reversion of the expression of Bcl-2 and Bax	(215)
			Doxorubicin-induced H9c2 cells	Protection against ER stress; downregulation of the expression of ER stress marker proteins; ER stabilization through the activation of the SIRT1 pathway	(216)
	Parthenolide	Migraine; arthritis; AS; ischemic injury in brain; cancer	Jurkat cell	Promotion of plaque stability; decrease of NF-κB activation and FasL expression	(102)
			Permanent MCAO rat model	Downregulation of NF-κB, phosho-p38 MAPK, and caspase-1 expression	(220)
	Reticuline	Cardiovascular diseases and inflammatory diseases	Xylene-induced ear edema and carrageenan-induced paw edema in mice and rats	Inhibition on the expression of pro-inflammatory cytokines, such as TNF-α and IL-6; targeting JAK2/STAT3 and NF-κB pathway	(221)
	Sappanone A	Inflammatory diseases	LPS-stimulated RAW264.7 macrophages	Induction of HO-1 expression by activating Nrf2 through the p38 MAPK pathway	(222)
	Isoliquiritigenin	Cancer; infection; inflammatory and neurological diseases	Collagenase IV-induced intracerebral hemorrhage rat model	Suppression of ROS- and/or NF-κB-mediated NLRP3 inflammasome activation by promoting Nrf2 antioxidant pathway	(223)
NcRNAs	Mir-181a-5p/3p	Vascular inflammation and AS	ApoE knock-out mice	Alleviation of atherosclerotic plaque formation; decrease of proinflammatory gene expression; decrease of infiltration of macrophage, leukocyte and T cell into the lesions; targeting TAB2 and NEMO	(224)
	LncRNA VINAS	AS	LDLR knock-out mice	VINAS knockdown reduces atherosclerotic lesion formation and expression of key inflammatory markers and leukocyte adhesion molecules; targeting MAPK and NF-κB signaling pathway	(225)
	LncRNA NORAD	Cancer; AS	OxLDL-treated HUVECs and high-fat-diet ApoE knock-out mice	Increase of endothelial viability; targeting NF-κB, p53-p21 signaling pathways and IL-8	(226)
	Circ-Sirt1	Cardiovascular diseases	HUVECs, human and rat VSMCs	Inhibition on inflammatory phenotypic switching of VSMC and neointimal hyperplasia; impeding NF-κB translocation and its binding to DNA	(85)

ERK, extracellular signal-regulated kinase; JAK, Janus kinase; OSCC, oral squamous cell carcinoma; Bax, Bcl2-Associated X; Bad, Bcl-2 associated death promoter; Bcl-2, B-cell lymphoma 2; ET, endothelin; mTOR, mammalian target of rapamycin; BI-1, Bax inhibitor-1; NSCLC, non-small-cell lung cancer; HUVEC, human umbilical vein endothelial cell; eNOS, endothelial nitric oxide synthase; MCAO, middle cerebral artery occlusion.

### NF-κB inhibitors

BAY 11-7082 (BAY) inhibits IKK-mediated phosphorylation of IκBα, resulting in decreased NF-κB and decreased expression of adhesion molecules. In addition, BAY also suppresses the translocation and activation of AP-1, interferon regulatory factor-3 (IRF-3), and signal transducer and activator of transcription-1 (STAT-1) by inhibiting the phosphorylation or activation of ERK, p38, and JAK-2 (192). BAY is also an inhibitor of NLRP3 inflammasome and a modulator of apoptosis pathways shown in the management of psoriasis-like dermatitis and oral cancer (193, 194). These suggest that BAY could serve as a lead compound in developing potent anti-inflammatory drugs with multiple targets in inflammatory responses.

Pyrrolidine dithiocarbamate (PDTC), another NF-κB inhibitor, leads to PDTC-dependent VSMC growth inhibition by inducing marked activation of p38 MAPK and JNK (195). In addition, PDTC blocks IL-18 signaling in ApoE knock-out mice, thus reducing inflammation and restoring plaque instability (196). A better understanding of the molecular mechanisms of PDTC provides a theoretical basis for clinical applications of antioxidants in AS.

IMD-0354 is an IKKβ inhibitor known to exert anti-inflammatory, antitumor, and radioprotective effects. The NF-κB activation induced by TNF-α and associated up-regulation of endothelin B2 receptor could be effectively suppressed by IMD-0354 in VSMCs (197). Additionally, IMD-0354 is confirmed as a potent inhibitor of glutamine uptake that concomitantly attenuates mTOR signaling, but not IKK-NF-κB signaling, suppresses the growth of melanoma cells, and induces autophagy and apoptosis. Affected genes and molecules are implicated in ROS/UPR signaling, including ATF4 and CHOP (198). IMD-0354 has been applied in phase I clinical trials for atopic dermatitis and choroidal neovascularization, though its cardiovascular protective effect has not been verified in clinical trials.

Blockage of NF-κB alone might be insufficient for AS mitigation. Combination with NF-κB inhibitors and lipid-regulating drugs such as statins could be a feasible scheme. Considering that persistent NF-κB inhibition could cause immune deficiency, future NF-κB inhibitors for AS treatment should only be used as adjuvant and intermittent medicine. In a word, the diversity of NF-κB modification signals makes it a long way to apply NF-κB inhibitors in anti-atherosclerotic therapy.

### Unfolded protein response inhibitors

Given the associations mentioned above between the UPR and NF-κB, the new functions of UPR inhibitors deserve to be reconsidered. Three representative molecules are listed in Table 3, with a special focus on their influences on PERK/eIF2α, ROS production, and NLRP3 inflammasome activation. Sirtuin-1 (SIRT1), an NAD^+^-dependent deacetylase, protects cardiomyocytes from ER stress-induced apoptosis by attenuating PERK/eIF2α pathway activation (199). A myokine, irisin, inhibits the PERK/eIF2α/CHOP and ATF6/CHOP pathways and alleviates the apoptosis of macrophages induced by oxLDL (200). Mouse models have shown that irisin promotes endothelial cell proliferation and significantly reduces AS in mice by upregulating the expression of miRNA126-5p (201). In the last decade, abundant clinical studies on the protective functions of irisin in the cardiovascular system have made breakthroughs. A recent cohort study has indicated low serum irisin levels as biomarkers of subclinical AS (202). However, existing studies mainly focus on serum irisin level increase after beneficial interventions such as simvastatin or Omega-3 fatty acids, and direct clinical evidence is necessary before irisin application (203, 204). Still, irisin has a promising preventive and therapeutic prospect for AS. In mouse models, small molecules STF-083010 and 4μ8C have shown a role in reducing atherosclerotic plaque size by inhibiting IRE1α RNase activity, lipid-induced mtROS production, and NLRP3 inflammasome activation (205).

Although people already have much knowledge of UPR and its roles in the development of AS, clinical trials evaluating UPR inhibitors are still scanty. Considering that adaptive UPR is important for the recovery of ER homeostasis, UPR inhibition is possibly only an incidental anti-atherogenic mechanism for potential UPR inhibitor drugs. For clinical use, specific inhibition of critical interaction between NF-κB and ER stress in one checkpoint of UPR branches could be an optimal strategy.

### Reactive oxygen species-interfering molecules

Many molecules present with anti-oxidant activities are promising anti-atherogenic drugs. (E/Z)-BCI hydrochloride (BCI), a small molecule inhibitor of dual-specificity phosphatase 6 (DUSP6), activates the Nrf2 signaling pathway and inhibits NF-κB activity, alleviating inflammatory response and decreasing ROS production in LPS-activated macrophages (206). Dihydrolipoic acid exhibits strong antioxidant activities in many conditions, especially neuroinflammation and provides protection *via* Nrf2/HO-1/ROS/NLRP3 signaling cascade in LPS-induced behavioral deficits in rats (207). Novel CHOP activator LGH00168 inhibits the NF-κB pathway and induces ROS-mediated ER stress, leading to necroptosis in A549 human lung cancer cells (177).

Reactive oxygen species is an identified risk factor for cardiovascular diseases. The activation of UPR branches, especially IRE1α and PERK, leads to the abrogation of ER stress-generated ROS, thus alleviating endothelial dysfunction. As discussed later, many natural compounds work by mediating ROS generation. Physical exercise is regarded as a supplement to pharmacotherapy for cardiovascular diseases by reducing ER stress and ROS (208, 209). In conclusion, numerous pathways upstream of ROS make interventions on ROS one of the most prospective strategies in extensive clinical settings more than AS. One limitation of the clinical application of ROS-interfering small molecules is toxicity.

### Natural compounds

Baicalin is a primary active substance from the *Scutellaria* root and attenuates ER stress-related apoptosis *in vivo* mediated by CHOP/eNOS signaling pathway (210). Baicalin is a marketed drug in China for the treatment of hepatitis, but more convincing clinical outcomes are required to evaluate its efficacy in treating AS. Quercetin existing in the pericarp, flower, leaf, and seed of various plants has an effect on maintaining ER protein homeostasis probably by increasing ATF6 and reducing CHOP and GRP78 in glucosamine-induced macrophages (211). Quercetin has been applied in Phase 2/3 clinical trials on coronary artery disease, venous thromboembolism, hypertension, and heart failure, and assessed as disease improvement effects (212–214). Resveratrol found in red wine attenuates cardiomyocyte hypertrophy and apoptosis in isoproterenol-induced rat cardiomyocytes, characterized by a low level of GRP78, GRP94, and CHOP, and by a reversed level of Bcl-2 and Bax (215). Resveratrol also alleviates doxorubicin-induced cardiocyte apoptosis of rats by relieving ER stress-related inflammatory response and activating SIRT1 signaling (216). A series of clinical studies have shown that dietary resveratrol improves endothelial function and exerts a beneficial effect on AS (217–219). Parthenolide is demonstrated to be an anti-inflammatory mediator and an NF-κB inhibitor, which has a potential application in cardiovascular and cerebrovascular diseases. Studies have demonstrated that the NF-κB/FasL signaling contributing to plaque rupture could be inhibited by parthenolide (102). Furthermore, the neuroprotective effect of parthenolide is characterized by the downregulation of NF-κB, phospho-p38 MAPK, and caspase-1 (220). Reticuline has anti-inflammation roles in CVDs by targeting the JAK2/STAT3 and NF-κB pathway, though the specific mechanisms are still unknown and further verification in atherosclerotic models is required (221). Sappanone A increases the level of HO-1 mediated by p38/Nrf2 signaling and suppresses LPS-induced NF-κB activation by modulating the p65 subunit, indicating its anti-inflammatory effect (222). Isoliquiritigenin from *Glycyrrhiza glabra* could reduce early neuronal degeneration after intracerebral hemorrhage, involving the NLRP3 inflammasome regulated by ROS and/or NF-κB through inducing Nrf2-mediated antioxidant activity (223).

The health effects of natural compounds in humans are limited by their purity and poor bioavailability, as they are extracted from plants and rapidly metabolized and excreted. Nevertheless, due to their easy availability from daily meals, diet change could be a simple and beneficial intervention. We can assume that natural compounds have a very high application value in AS prevention and treatment as well as improvement of general health conditions.

### NcRNAs

NcRNAs have received most and more attention over the last decades for their involvement in the progression of AS. Research has identified two microRNAs, miR-181a-5p and miR-181a-3p, cooperatively recede endothelium inflammation through blockade of the NF-κB signaling pathway by post-transcriptional regulation of TAB2 and NEMO expression, respectively (224). Long ncRNA (lncRNA) VINAS is highly expressed in intimal AS lesions and promotes vascular inflammation by a possible mechanism involving MAPK and NF-κB signaling pathways. Knockdown of lncRNA VINAS decreases the expression of adhesion molecules such as E-selectin, VCAM-1, and ICAM-1 and inflammatory molecules such as MCP-1, TNF-α, IL-1β, and COX-2 (225). LncRNA NORAD (non-coding RNA activated by DNA damage) knockdown aggravates oxidative stress, increases phosphorylated IκBα level and NF-κB nuclear translocation, and directly promotes IL-8 transcription in AS model. Therefore, lncRNA NORAD has a role in attenuating endothelial cell injury and alleviating AS (226). In contrast, ncRNA circ-Sirt1 directly binds to NF-κB and inhibits its translocation (85).

A number of RNA therapeutics have been in clinical phase II or III for various diseases, but lncRNAs are not among them. Moreover, up to now, few RNA therapies have been explored for cardiovascular diseases. The application of ncRNA therapeutics in AS requires overcoming many challenges, including immunogenicity, lack of specificity, and delivery difficulty.

## Conclusion

As NF-κB and ER stress are involved in many human physiological processes, such as immunity and cancer, there are certain limitations to be overcome before therapeutically targeting them in AS. Also, new drug development is limited by the complexity of intrinsic pathways and crosstalk with other pathways. Therefore, the unexpected effects should be considered with caution when evaluating the safety of NF-κB and ER stress as targets for treatment. In this regard, it is significant to further explore more specific and effective crosstalk inhibitors and/or enhancers for atherogenesis, while leaving the normal physiological functions unaffected. On the other hand, these crossover effects also mean that a single successful drug may have utility in multiple diseases.

Indeed, currently available studies provide only a theoretical prospect of targeting interactions between NF-κB and ER stress against AS, and more convincing experiments are required to come closer to the production of an effective NF-κB targeting anti-atherogenic drug. Nevertheless, a broader and deeper understanding of NF-κB signaling and recognition of the potential direct or indirect links between these divergent pathogenic processes may eventually define the value of targeting their crosstalk as a clinical application to AS.

## Author contributions

WL, KJ, JL, and WX contributed to the conception, reviewed for important intellectual content, and wrote the majority of the text and created the figures. YJW, JZ, YLW, and RX provided some text. LJ, TW, and GY edited the manuscript. All authors read and approved the final manuscript.
